# Extramedullary Manifestation in Multiple Myeloma Bears High Incidence of Poor Cytogenetic Aberration and Novel Agents Resistance

**DOI:** 10.1155/2015/787809

**Published:** 2015-04-23

**Authors:** Xiaoyan Qu, Lijuan Chen, Hairong Qiu, Hua Lu, Hanxin Wu, Hongxia Qiu, Peng Liu, Rui Guo, Jianyong Li

**Affiliations:** Department of Hematology, Jiangsu Province Hospital, First Affiliated Hospital of Nanjing Medical University, 300 Guangzhou Road, Jiangsu, Nanjing 210029, China

## Abstract

Extramedullary disease (EMD) in multiple myeloma (MM) patients is an uncommon event and more attention was directed toward the feature of these patients. Cytogenetic aberration is an important characteristic of MM and is associated with patients' outcome. In this study, we aimed to compare the cytogenetic abnormality of patients with and without extramedullary manifestation, and to analyze the clinical outcomes of novel agents in EMD patients. We retrospectively investigated data from 41 MM patients. Our analyses showed del(17p13) in 31% of EMD versus 13% of medullary disease (*P* = 0.03) and amp(1q21) in 55% versus 32% (*P* = 0.019). No differences were shown in del(13q14) and t(4;14). 24/27 patients with EMD at diagnosis responded to the novel agents-containing regimens. However, when relapsed, 70% of patients did not benefit from the sequential use of novel agents as salvage therapy. In 14 patients who developed EMD at relapse phase, only 2 patients responded to novel agents therapy. Median overall survival of patients with extramedullary manifestations was 30 months, in comparison to 104 months for patients without EMD (*P* = 0.002). Patients with extramedullary manifestation bore high incidence of poor cytogenetic aberration and novel agents resistance.

## 1. Introduction

Multiple myeloma (MM) is a clonal B-cell malignancy characterized by the aberrant proliferation of plasma cells within the bone marrow (BM). However, the disease typically remains confined to the BM [1]. A small number of patients develop extramedullary disease (EMD) at diagnosis, at progression, or during relapse phase.

The reported incidence of EMD in newly diagnosed MM varies from 7% to 18%. Moreover, 6% to 20% of patients develop EMD later in the course of the disease [2–5]. In the past, extramedullary relapse (EMR) was uncommonly encountered in clinic and is not described that often owing to the short life expectancy of patients with MM. Due to the more sensitive imaging techniques and the prolonged patients' survival, the incidence of EMD during disease course is rising [2]. Thus, more attention was directed toward the patients with EMD.

In the case of MM patients, EMD can be present at the time of initial diagnosis or can develop at the time of relapse. Based on the published literature, EMD is a poor prognostic marker in both newly diagnosed and relapsed MM patients and, therefore, is a therapeutic challenge [6–8]. Even in the era of novel agents, EMD was still associated with poor outcome in patients. Increasing EMD cases resistant to novel targeted agents were reported [9–11].

Fluorescence in situ hybridization (FISH) and conventional cytogenetic (CC) studies serve as the cornerstone of risk stratification in MM. These methods are used to distinguish patients who will have an aggressive course and are resistant to therapies from those whose disease will be indolent and slow to relapse [12]. CC abnormalities are seen in a minority of patients with MM because of slow division of neoplastic plasma cells; however, interphase FISH assay is independent of the plasma cell division and has a higher yield to detect genetic aberrations [13]. The deletion of p53 (locus 17p13), immunoglobulin heavy chain (IgH) translocations, t(4;14) (p16.3;q32), t(11;14) (q13;q32), t(14;16) (q32;q23), and 1q21 amplification[amp(1q21)] are more commonly applied for stratification. The t(4;14) abnormality (associated with fibroblast growth factor receptor 3 expression) is detected in approximately 10% to 15% of patients. These patients have an intermediate risk status and tend to be more responsive to bortezomib based therapy [12]. The t(11;14) (q13;q32) is present in up to 20% of patients and confers a favorable prognosis [14]. The tumor suppressor gene, p53, resides at 17p13 locus, and its loss confers survival disadvantage, irrespective of whether novel agents were used [15]. The amp(1q21) is considered a high risk feature and confers bortezomib resistance [16].

Patients with EMD at diagnosis or during disease course were associated with targeted drug resistance. However, whether the adverse effect of EMD on patients was related to cytogenetic aberrations remained unclear. There are only a few studies demonstrating the cytogenetic aberrations in myeloma patients with EMD [17–19]. In this study, we aimed to compare the cytogenetic abnormality of MM patients with EMD and patients without extramedullary manifestations and to analyze the clinical outcomes of novel agents in MM patients with EMD.

## 2. Materials and Methods

### 2.1. Patients

We screened our MM database for patients treated at the First Affiliated Hospital of Nanjing Medical University between December 2007 and May 2014 who either presented with EMD at diagnosis or developed EMD at disease progression or relapse. The study has been approved by the Ethics Committee of the First Affiliated Hospital of Nanjing Medical University. Written informed consent was obtained from all human participants.

The EMD was defined as the presence of plasma cell tumor outside the bone marrow, either in the form of soft tissue mass or skeletal EMD, with plasma cell tumors spreading from bone disease or arising in extraosseous organs. The EMD was diagnosed using imaging methods, such as computed tomography (CT), magnetic resonance imaging (MRI), or PET/CT. Biopsies confirmation was carried out whenever possible and the proof of invasion of central nervous system (CNS) was ascertained by positive cytologic findings in cerebrospinal fluid.

Durie and Salmon criteria were used for diagnosis and staging [20]. The International Staging System was also applied to patients [13]. All of the patients received novel agents-based (thalidomide, lenalidomide, and bortezomib) therapy: TAD (thalidomide, doxorubicin, and dexamethasone), TCD (thalidomide, cyclophosphamide, and dexamethasone), TD (thalidomide and dexamethasone), PAD (bortezomib, doxorubicin, and dexamethasone), PCD (bortezomib, cyclophosphamide, and dexamethasone), VTD (bortezomib, thalidomide, and dexamethasone), VD (bortezomib and dexamethasone), MPT (melphalan, prednisone, and thalidomide), RD (lenalidomide and dexamethasone), VAD-T (vincristine, doxorubicin, dexamethasone, and thalidomide), and MOD-T (mitoxantrone, vincristine, dexamethasone, and thalidomide). In this analysis, complete response (CR), very good partial response (VGPR), partial response (PR), stable disease (SD), progressive disease (PD) status, and clinical relapse were defined according to the International Myeloma Working Group Uniform Response Criteria [21].

### 2.2. Interphase Fluorescence In Situ Hybridization (FISH)

Interphase FISH was performed in all cases on bone marrow smears, as described previously [22]. The FISH panel included D13S319 and locus specific identifier (LSI) 13 (RB-1) probes for the detection of 13q14 deletion [del(13q14)] and a 1q21 (CKS1B) probe and 17p13.1 (P53) probe for detection of amp(1q21) and 17p13 deletion [del(17p13)], respectively. An LSI IGHC/IGHV dual-color, break-apart rearrangement probe was used to determine the translocations involving IgH; LSI IGH/CCND1, LSI IGH/FGFK3, and LSI IGH/MAF probes were further used to detect t(11;14) (q13;q32), t(4;14) (p16;q32), and t(14;16) (q32;q23) in patients with 14q32 rearrangement. Fluorescent images were captured with epifluorescence microscope (Leica DRMA2, Germany) equipped with CCD camera (AI Company, UK) and using appropriate filters. Two hundred nuclei were scored for each probe. Bone marrow cells samples of 10 cytogenetically normal individuals served as controls. The cut-off level for positive value of each probe in I-FISH was 10.0%.

### 2.3. Statistical Analysis

All statistical analyses were performed using SPSS 17.0 software. Kaplan-Meier curves for progression-free survival (PFS) and overall survival (OS) were plotted and compared by log-rank test. The statistical significance of differences in clinical characteristics between patients was assessed using the *χ* ^2^ test. A *P* value < 0.05 was considered to be statistically significant.

## 3. Result

### 3.1. Patient Characteristic

Forty-one MM patients with EMD involvement were collected in this study. Two patients evolved from solitary plasmacytomas. We found evidence of EMD in 9% (27 of 300) of newly diagnosed patients (EMD-1) (Table 1). Fourteen patients developed EMD in the course of the disease (EMD-2) (Table 2): 8 patients (57%) at first relapse and 6 patients (43%) at second and higher relapse. There were 27 men and 14 women with EMD myeloma. The median age was 58 years (range 39–78) of MM patients. According to the Durie-Salmon (D-S) staging system [20], one patient was stage I, three patients were stage II, and the remaining 37 patients were stage III. According to the ISS staging system [13], 14 patients were stage I, 15 patients stage II, and the remaining 12 patients stage III. The monoclonal component was of IgG type in 21 cases, IgA type in 12 cases, IgM type in one case, and light chain type in 7 cases. The result of immunohistochemistry for extramedullary involvement was CD38^++^, CD138^++^, CD20^+/−^, CD56^−^, and ki-67 20%–50%^+^.

**Figure 1:**
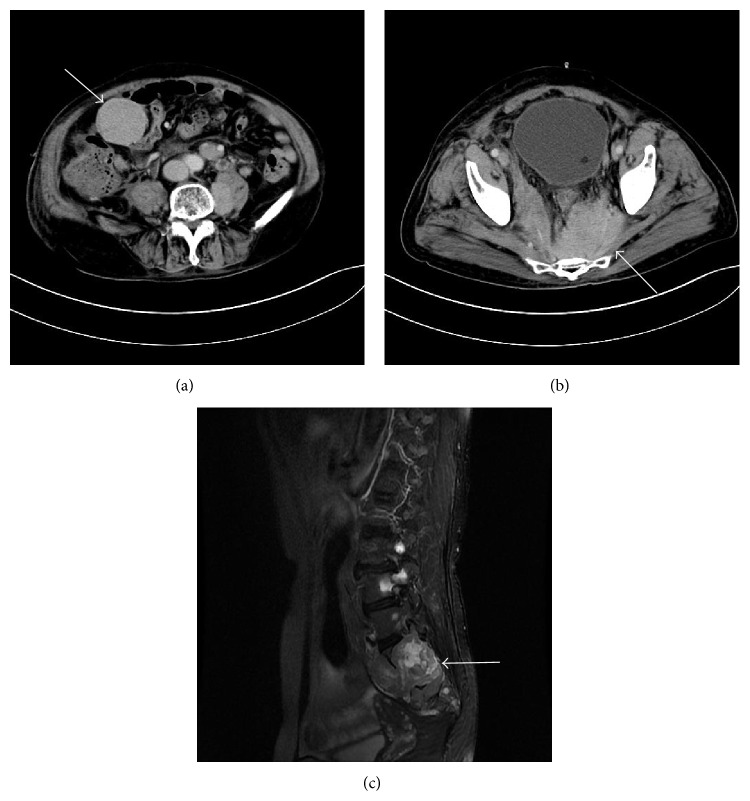
CT/MRI scan images. (a) Enhanced abdominal CT demonstrated soft tissue mass in the right middle abdomen (arrow). (b) Enhanced abdominal CT demonstrated soft tissue mass in the pelvic area (arrow). (c) Fat-suppressed T2-weighted lumbar spine image showed mass (arrow) and the vertebral body and appendix are involved.

For 14 patients who had developed EMD during disease progression or relapse, the median interval between diagnosis of MM and diagnosis of EMD myeloma was 16.5 months; the longest interval was 70 months. The main sites involved in patients with EMD were the soft tissues (25/41 patients, 61%). Other sites included the lymph nodes (3 cases), liver (2 cases), CNS (4 case), skin (2 case), pelvic area (1 case), bone (8 case), and abdominal cavity (1 case). Four patients with EMD indicated involvement at more than one site. The representative CT/MRI scans of two patients with EMD are depicted in Figure 1.

### 3.2. FISH Results

Among the 41 bone marrow aspirates, 29 were assessable for cytogenetics analysis (lack of plasma cells or FISH failure in 12 samples). In EMD-1 group, baseline cytogenetics were available in 78% of the patients (21/27): del(17p13) in 29% (6/21), del(13q14) in 57% (12/21), amp(1q21) in 57% (12/21), and t(4;14) in 19% (4/21). In EMD-2 group, molecular cytogenetics from initial MM diagnosis were available in 8 patients: del(17p13) in 3 patients, del(13q14) in 4 patients, and amp(1q21) in 4 patients. No patients harbored t(4;14) or t(11;14). In both groups, no patient had t(14;16).

We also analyzed the incidence of cytogenetic aberration in patients without EMD treated in our hospital between December 2007 and May 2014. Molecular cytogenetics were available in 134 patients: del(17p13) in 13% (17/134), del(13q14) in 45% (60/134), amp(1q21) in 32% (43/134), t(11;14) in 21% (28/134), t(4;14) in 12% (16/134), and t(14;16) in 4% (5/134). No differences were shown in the incidence of del (13q14) and t(4;14) between EMD myeloma patients and medullary myeloma patients. However, the incidences of del(17p13) and amp(1q21), when compared with medullary myeloma, are significantly higher (*P* = 0.03 and 0.019, resp.). We did not detect t(11;14) or t(14;16) in patients with EMD.

### 3.3. Prognosis and Response to Therapy

**Figure 2:**
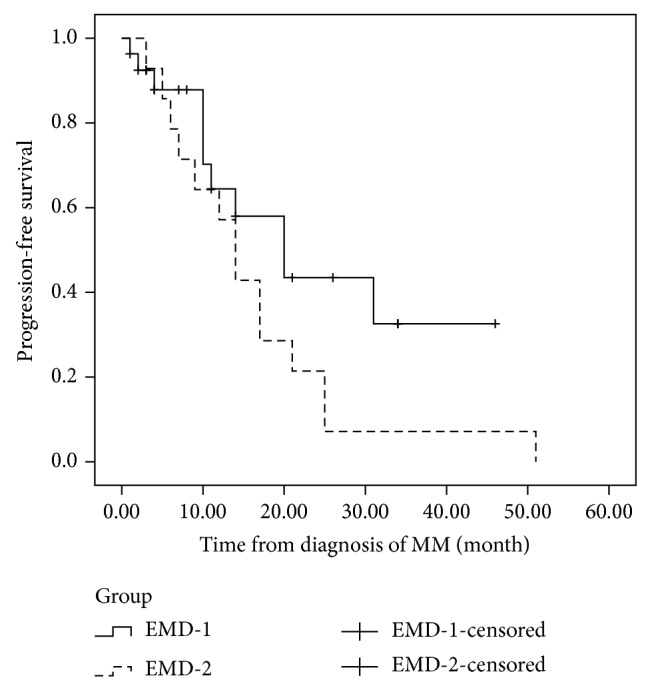
The progression-free survival (PFS) of patients with extramedullary disease at diagnosis and at relapse. The median duration of PFS of patients in EMD-1 and EMD-2 was 20 months and 14 months, respectively (*P* = 0.114).

**Figure 3:**
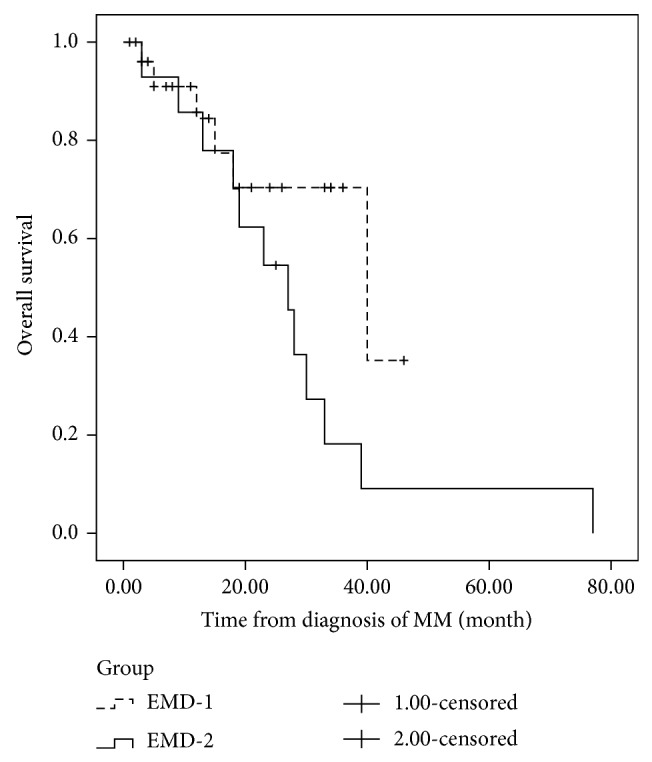
The overall survival (OS) of patients with extramedullary disease at diagnosis and at relapse phase. The OS of patients with extramedullary involvement at initial diagnosis and patients experiencing extramedullary disease at relapse phase was 40 months and 27 months, respectively. No difference was shown in the OS between the two groups (*P* = 0.076).

In EMD-1 group, the regimens used for the initial treatment were TAD (9 patients), TCD (1 patient), TD (2 patients), PAD (3 patients), PCD (5 patients), VTD (3 patients), VD (2 patients), MPT (1 patient), and RD (1 patient). In this group, only one patient received autologous stem cell transplantation. All of these patients received a novel agents-included therapy. After induction therapy, 24/27 (89%) patients responded to the novel agents-containing regimens (CR, VGPR, or PR), and the complete response was 19%. As of July 1, 2014, 10 patients had relapsed. Moreover, all of these patients presented extramedullary relapse with coexisting bone marrow relapse. New agents-based therapy has been used in the relapse setting. However, only 2 patients responded to bortezomib-based therapy and 1 patient responded to lenalidomide and dexamethasone. After a median followup of 14 months (range 5–46 months) from diagnosis, the median duration of PFS of patients was 20 months (Figure 2) and the median OS was 40 months (Figure 3).

**Figure 4:**
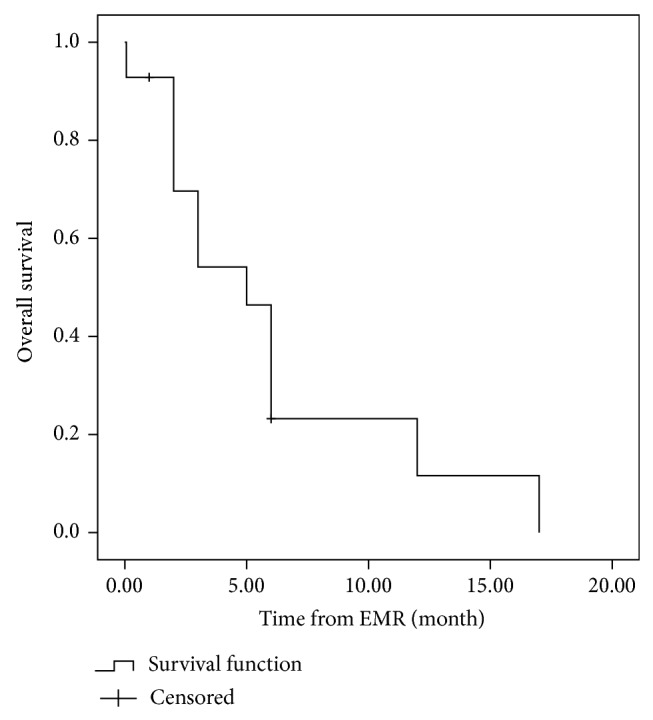
The overall survival (OS) of patients after extramedullary relapse. Median OS of patients in EMD-2 from extramedullary relapse was only 5 months.

**Figure 5:**
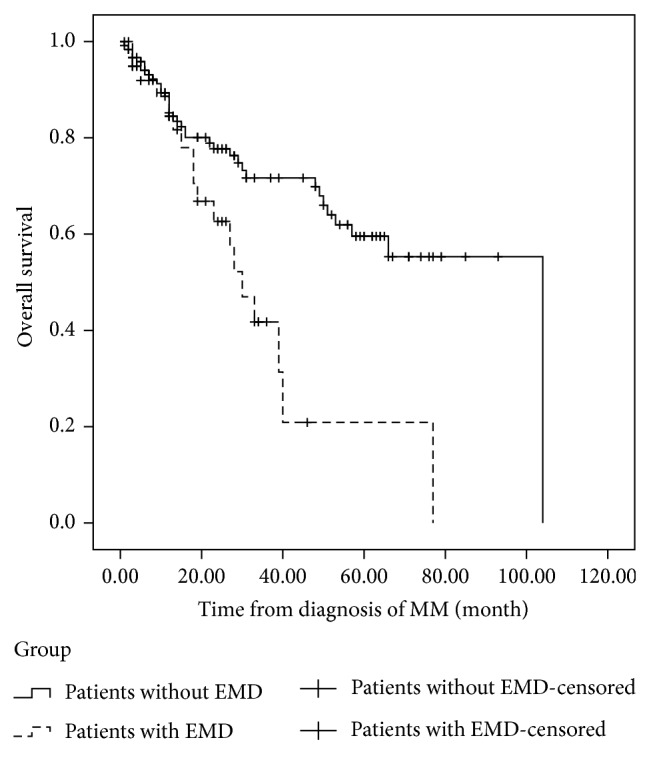
The overall survival (OS) of patients with extramedullary disease (EMD) and patients without extramedullary involvement. In 134 patients without EMD assessable for cytogenetics, 11 patients were lost to followup. The median OS of 123 patients without EMD was 104 months, in comparison to 30 months for patients with EMD involvement (*P* = 0.002).

In EMD-2 group, the regimens used for the initial treatment were VTD (4 patients), TAD (1 patient), VAD-T (4 patients), TD (1 patient), VD (1 patient), MPT (1 patient), MOD-T (1 patient), and PCD (1 patient). The median interval between diagnosis of MM and EMR was 16.5 months (range 3–70 months). After a median followup of 24 months (range 3–77 months) from diagnosis, the median PFS was 14 months (Figure 2). After extramedullary relapse, bortezomib-based regimens were performed on 7 patients, thalidomide-based therapy had been given to 2 patients, and lenalidomide-based regimens were used in 3 patients. One patient received radiotherapy only. One patient refused further therapy. Only two patients who received lenalidomide and dexamethasone achieved further response. However, the duration of response of both patients was short. The OS after EMR of responding patients was only 12 and 17 months, respectively. These patients with EMR bore potential therapeutic difficulties and novel agents resistance. The median OS from diagnosis and from EMR was only 27 months (Figure 3) and 5 months (Figure 4), respectively. However, no differences were shown in the PFS (*P* = 0.114) or OS (*P* = 0.076) between patients with EMD at diagnosis and patients experiencing EMD at relapse phase. We also compared the OS of patients with EMD and patients without EMD. In 134 patients without EMD assessable for cytogenetics, 11 patients were lost to followup. The median OS of 123 patients without EMD was 104 months, in comparison to 30 months for patients with EMD involvement (*P* = 0.002) (Figure 5).

## 4. Discussion

In this retrospective study, we describe 41 patients with EMD myeloma encountered over the past 7 years at our hospital from 300 patients with MM. The incidence was 14%, which is similar to previous reports [2–5]. Due to the more sensitive imaging techniques and the prolonged patients' survival, the incidence of EMD during disease course is rising [2]. EMD MM, especially EMR, appears to be an uncommon but important phenomenon and needs more emphasis to be put on.

EMD MM appears to have a specific clinical manifestation. The analysis of the presenting features of EMD MM shows they are significantly distinct from the rest of the MM population concerning age, sex, MM subtype, disease stage, and prior history of MGUS [2]. In addition, the disease course is presented differently from patients without EMD. Varettoni et al. [2], using a time-dependent analysis, demonstrated that presence of extramedullary involvement at any time in the course of disease was associated with significantly shorter PFS and OS. Patients presenting EM involvement at diagnosis had significantly shorter PFS as compared with the rest of MM population (18 versus 30 months). A retrospective single-center study of 24 cases demonstrated the median PFS was 2 months and the median OS was 7 months after diagnosis of EMR [7]. Fassas et al. [23] reported the median OS from the time of diagnosis of CNS involvement was only 1.5–2 months.

Even in the era of novel agents, EMD was associated with poor prognosis and drug resistance [6]. Rosiñol et al. [10] reported that none of 11 patients with EMD responded to single-agent thalidomide, as compared with 16 responders among 27 patients without extramedullary involvement. Although 4 of the 11 patients with extramedullary involvement had a serological response, a progression of the soft tissue masses was observed in all of them. Another study also showed, in patients with extramedullary involvement, the use of thalidomide did not improve outcome [3]. With regard to bortezomib efficacy in EMD MM, several case reports showed that patients with EMD responded to bortezomib-based regimens [24, 25]. However, the number of patients studied at present has been small. Bortezomib has extensive tissue penetration; however, data from studies conducted in nonhuman primates have demonstrated that bortezomib cannot penetrate into the CNS or into various regions of the eye [26]. In this study, all of the 27 patients having EMD involvement at diagnosis received novel agents-containing therapy. Three patients receiving thalidomide-containing regimens responded poorly to the induction therapy. Ten patients in EMD-1 group had experienced relapse with EMD. However, only 3 patients benefited from the sequential use of novel agents as salvage therapy. Two patients responded to bortezomib-based therapy and 1 patient responded to lenalidomide and dexamethasone. We treated all of the patients who developed EMD at relapse phase with novel agents-containing therapy. However, only 2 patients obtained short response after lenalidomide-containing therapy. The remaining patients had novel drugs resistance and did not achieve further response.

Hitherto there is no consensus about the best therapeutic choice for EMD patients. In this study, 89% (24/27) patients having EMD involvement at initial diagnosis responded to novel agents-based therapy. When patients developed EMD involvement at relapse phase, 75% (9/12) of patients presented novel agents resistance. Data on the prognostic factors which impact the response of EM involvement in MM are limited. Cytogenetic abnormalities are considered useful factors for prognostication of patients with MM. A series of studies have indicated that patients with t(4;14) may benefit from use of bortezomib, either as induction therapy or long-term treatment [15, 27, 28]. In this study, we did not find differences of t(4;14) between EMD patients and patients without EM involvement. Translocation t(11;14) (q13;q32), which is found in about 15% of patients, appears to be associated with a favorable outcome and therefore is considered neutral with regard to prognosis [29, 30]. However, there was no patient harboring t(11;14) in our study. Due to the low incidence of translocation t(14;16), large series of cases are further needed to confirm the incidence of this abnormality in patients with EMD. Patients with del(17p13) were defined as having high-risk disease; no specific treatment has so far demonstrated a beneficial effect [15]. Two studies demonstrated the incidence of del(17p13) in EMD patients was significantly higher than that in the patients without EMD reported by the published literature data [7, 17]. In this study we compared the incidence of del(17p13) between patients with EMD and patients without EMD. The result showed that the incidence was higher. Also, chromosome 1 amplification was considered an indicator of poor outcome even in the use of novel regimens [31]. To the best of our knowledge, there was no report that studied the chromosome 1 aberration in patients developing EMD. We detected that the incidences of amp(1q21) were also higher in EMD patients when compared to medullary disease. Thus, we think this incidence difference of poor cytogenetic aberration may be one of the causes of novel agents resistance.

## 5. Conclusions

MM with EM involvement, especially EMR, appears to be an uncommon but important phenomenon. EMD MM appears to have a specific clinical manifestation. In this study, we have demonstrated 41 cases of MM patients presenting extramedullary manifestation. These MM patients confer higher incidence of del(17p13) and amp(1q21) and potential therapeutic difficulties. Patients with extramedullary relapse pattern were resistant to novel targeted agents and were associated with poor prognosis. Further studies are needed to explore the optimal therapeutic strategies to deal with the phenomena.

## Figures and Tables

**Table 1:** Clinical and FISH data of MM patients with EMD at initial diagnosis.

*n*		27
Age (median) range in years		60 (39–78)

Sex (male/female)		16/11

Stage (Durie-Salmon)	I	1
	IIA	2
	IIB	1
	IIIA	19
	IIIB	4

Stage (ISS)	I	10
	II	10
	III	7

MM type	IgG	16
	IgA	6
	Light chain	5

FISH result	del(17p13)	6
	del(13q14)	12
	amp(1q21)	12
	t(4;14)	4
	Not available	6

Involved sites	Soft tissues	17
	Lymph nodes	3
	Bone	8
	Abdominal cavity	1
	Pelvic area	1

**Table 2:** Clinical and FISH data of MM patients with EMD at relapse stage.

*n*		14
Age (median) range in years		58 (39–78)

Sex (male/female)		11/3

Stage (Durie-Salmon)	IIIA	11
	IIIB	3

Stage (ISS)	I	4
	II	5
	III	5

MM type	IgG	5
	IgA	6
	IgM	1
	Light chain	2

FISH result	del(17p13)	3
	del(13q14)	4
	amp(1q21)	4
	Not available	6

Involved sites	Soft tissues	8
	Central nervous system	4
	Skin	2
	Liver	2

Median time to EMR (month)		16.5 (3–70)
